# Deciphering Single Nucleotide Polymorphisms and Evolutionary Trends in Isolates of the Cydia pomonella granulovirus

**DOI:** 10.3390/v9080227

**Published:** 2017-08-18

**Authors:** Jörg T. Wennmann, Pit Radtke, Karolin E. Eberle, Gianpiero Gueli Alletti, Johannes A. Jehle

**Affiliations:** 1 Institute for Biological Control, Federal Research Centre for Cultivated Plants, Julius Kühn Institute, Heinrichstraße 243, 64287 Darmstadt, Germany; joerg.wennmann@julius-kuehn.de (J.T.W.); Karolin.Eberle@ams-europe.com (K.E.E.); gianpiero.guelialletti@googlemail.com (G.G.A.); 2 Department of Phytopathology, Agricultural Service Center Palatinate (DLR Rheinpfalz), 67435 Neustadt an der Weinstrasse, Germany; Pit.Radtke@dlr.rlp.de

**Keywords:** *Cydia pomonella granulovirus*, codling moth, single nucleotide polymorphism, mutation, resistance

## Abstract

Six complete genome sequences of Cydia pomonella granulovirus (CpGV) isolates from Mexico (CpGV-M and CpGV-M1), England (CpGV-E2), Iran (CpGV-I07 and CpGV-I12), and Canada (CpGV-S) were aligned and analyzed for genetic diversity and evolutionary processes. The selected CpGV isolates represented recently identified phylogenetic lineages of CpGV, namely, the genome groups A to E. The genomes ranged from 120,816 bp to 124,269 bp. Several common differences between CpGV-M, -E2, -I07, -I12 and -S to CpGV-M1, the first sequenced and published CpGV isolate, were highlighted. Phylogenetic analysis based on the aligned genome sequences grouped CpGV-M and CpGV-I12 as the most derived lineages, followed by CpGV-E2, CpGV-S and CpGV-I07, which represent the most basal lineages. All of the genomes shared a high degree of co-linearity, with a common setup of 137 (CpGV-I07) to 142 (CpGV-M and -I12) open reading frames with no translocations. An overall trend of increasing genome size and a decrease in GC content was observed, from the most basal lineage (CpGV-I07) to the most derived (CpGV-I12). A total number of 788 positions of single nucleotide polymorphisms (SNPs) were determined and used to create a genome-wide SNP map of CpGV. Of the total amount of SNPs, 534 positions were specific for exactly one of either isolate CpGV-M, -E2, -I07, -I12 or -S, which allowed the SNP-based detection and identification of all known CpGV isolates.

## 1. Introduction

The Cydia pomonella granulovirus (CpGV) is a double-stranded DNA (dsDNA) virus that constitutes the type species *Cydia pomonella granulovirus* of the genus *Betabaculovirus* (family *Baculoviridae*) [1,2]. CpGV is highly infective to larvae of the codling moth (CM), *Cydia pomonella* L., a major pest in apple, pear and walnut plantations [3]. The host range of CpGV is limited to *C. pomonella* larvae and closely related Lepidopteran tortricids [4,5]. CpGV is used as an effective biological control agent for an environmental-friendly control of CM [3], with no effect on non-target insects and a high virulence causing rapid death of early larval instars [6]. To date, CpGV-based products are registered and applied in most apple-producing countries worldwide [5].

The first discovered isolate of CpGV was CpGV-M; its in vivo cloned genotype CpGV-M1 was the first completely sequenced CpGV isolate. Further CpGV isolates were isolated from infected CM larvae around the world, and are divided into five genome groups A to E, which represent phylogenetic lineages [5,7,8,9]. This classification is supported by single nucleotide polymorphisms (SNPs) in the *granulin* (*gran*) and *late expression factor 8* (*lef-8*) gene [7] and the concatenated amino acid sequences of 35 baculovirus core genes [5]. The five genome groups are represented by six entirely sequenced CpGV genomes [5,8]: (A) the Mexican isolate CpGV-M [9] and its in vivo cloned genotype CpGV-M1 [8]; (B) the English isolate CpGV-E2 [10]; (C) the Iranian isolate CpGV-I07; (D) the Iranian isolate CpGV-I12 [7,11] and (E) the Canadian isolate CpGV-S [5]. Partial sequences of further Iranian isolates CpGV-I01, -I08, -I66 and -I68 and Georgian isolates CpGV-G01 and -G02 are available at GenBank [7].

In the 1980s, CpGV-M was developed as an active agent of commercial European CpGV-based products for the control of codling moth [12]. New interest in other CpGV isolates was fueled by the emergence of CM field populations in Central Europe that were resistant to CpGV-M products [5,6,13,14]. For the field-derived CM strain CpRR1, it was demonstrated that resistance to CpGV was incompletely dominant, monogenetic, sex-linked inherited, and caused by an early block of virus replication [13,15]. A similar inheritance mode of CpGV resistance was assumed for French and Czech CM populations [14,16]. This form of resistance is considered to be type I resistance [17]. As shown by infection studies using different CpGV isolates, it is directed only against CpGV-M of genome group A, whereas CpGV isolates of genome group B to E appeared to be resistance-breaking [5,6]. Genome sequencing of these isolates revealed that CpGV-M differed from resistance-breaking CpGVs only in an additional 24 bp motif in the *pe38* gene, which appeared to be the target of type I resistance [5]. New strategies in the control of susceptible and resistant CM field populations comprised CpGV formulations that included resistance-breaking isolates; however, recent studies proposed a second type of CM field resistance (type II) that could not be controlled as anticipated by the new CpGV formulations [14,17,18].

The CpGV genome groups A to E represent the currently known genetic diversity of isolates constituting the species *Cydia pomonella granulovirus.* According to current definitions, the category of a virus species is defined as “a polythetic class of viruses that constitutes a replicating lineage and occupies a particular ecological niche” [19,20]. It is thus an assemblage of narrowly related isolates, which may differ in single characteristics such as nucleotide sequence, protein composition, virulence etc., yet share enough similarities to comply with this definition. Jehle et al. proposed a phylogenetic species concept of baculoviruses based on nucleotide sequence distances of highly conserved genes [1]. Though family-wide phylogeny and the evolution of baculoviruses had been intensively studied, the intra-species diversity of baculoviruses was only recently addressed [21,22,23].

After the genome sequencing of different CpGV isolates was performed to identify the major target of type I resistance [5], we had the unique opportunity to use the whole genome sequences for studying the diversity, phylogeny, and evolutionary trends within a baculovirus species. In particular, we focused on the detection of SNPs of the five entirely sequenced isolates to create a genome-wide map of genome group, and multiple genome group-specific SNP positions.

## 2. Materials and Methods

### 2.1. CpGV Isolates and Sequences

The genome sequences of six geographic isolates of CpGV were analyzed in this study: Mexican isolates (i) CpGV-M (KM217575) and (ii) CpGV-M1 (NC_002816), an in vivo cloned strain of CpGV-M; Iranian isolates (iii) CpGV-I07 (KM217574); and (iv) CpGV-I12 (KM217576); (v) Canadian isolate CpGV-S (KM217573), and (vi) English isolate CpGV-E2 (KM217577), an in vivo clone from the English field isolate CpGV-E [5].

### 2.2. Genome Sequencing

The isolate CpGV-I12 was Sanger sequenced at 3.9-fold coverage from a shotgun library (Table 1). The coverage was considered sufficient as major differences between CpGV-I12 and the reference genome CpGV-M1 [5] were already known by restriction length polymorphism (RFLP) analysis and partial sequencing [7]. The shotgun library was constructed from in total 60 µg viral DNA by shearing and subcloning average-sized 1200 bp. About 1000 clones were picked and Sanger sequenced. Primer walking was used to close occurring gaps.

About 10–20 µg of each CpGV-M, -S, -E2 and -I07 were sequenced according to the 454 pyrosequencing approach (Life Science/Roche, Brandfort, CT, USA), with an average 17.2-, 22.2-, 243- and 8-fold coverage, respectively (Table 1). Reads were assembled to contigs using SeqMan (Lasergene 8.0, Dnastar Inc., Madison, WI, USA). Contigs were fitted together manually or by re-assembling. Open reading frames (ORF) of CpGV-M1 that could be predicted in the newly sequenced isolates were annotated according to the CpGV-M1 sequence annotation [8]. Additional ORFs, which were at least 150 nt in length, did not overlap by more than 100 nt, and were not located within a homologous repeat regions (*hrs*), were added to the annotation. ORFs that violated these criteria but were identified as homologous baculovirus genes by BLAST (http://www.ncbi.nlm.nih.gov/BLAST/) were annotated as well. All library construction and sequencing steps were performed by StarSEQ company (Mainz, Germany).

### 2.3. Genome Analysis

For genome sequence analysis of CpGV-M, -M1, -I07, -I12, -S, and -E2, Geneious 8.1.9 software (Biomatters, Auckland, New Zealand) was used. The high co-linearity of genomes allowed the alignment of the entire genomes of all six isolates using Mauve alignment algorithm [24], as implemented in Geneious software. CpGV-M was set as the reference sequence. Repeated motifs were identified with REPuter [25]. If necessary, alignments were adjusted manually in repeat regions. SNPs were called in positions that were covered by the sequences of CpGV-M, -I07, -I12, -S and -E2.

### 2.4. Phylogenetic Analysis

CpGV-M1 was removed from the whole genome alignment, and the genome sequence of Cryptophlebia leucotreta granulovirus (CrleGV) (NC_005068) was included in the alignment. The obtained nucleotide alignment of CpGV-M, -I07, -I12, -S, -E2 and CrleGV was conducted for minimum evolution (ME) analysis using MEGA 5.2 software [26], with CrleGV as the outgroup. The phylogenetic tree was inferred by using 1000 bootstrap replicates with close-neighbor-interchange algorithm. An initial tree was obtained by neighbor joining.

## 3. Results and Discussion

**Figure 1:**
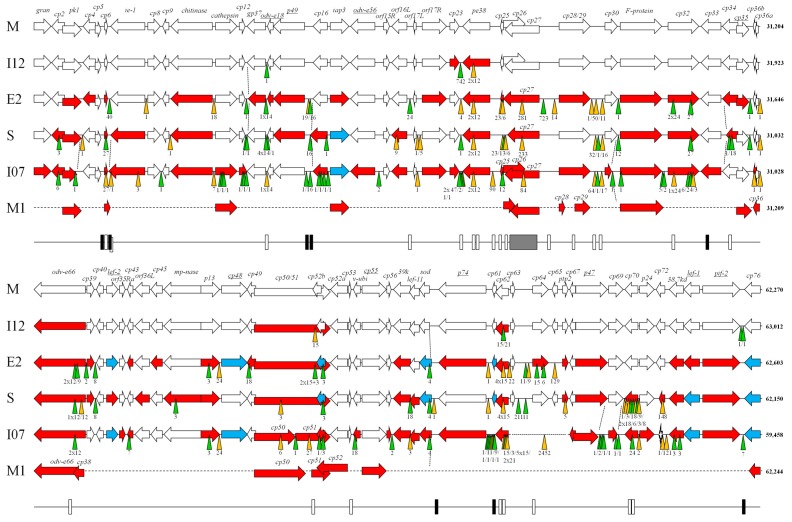

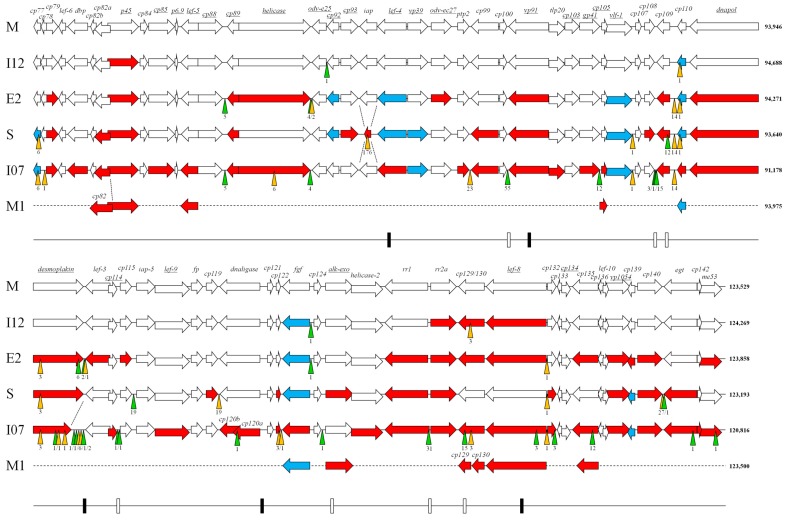
Graphical genome alignment of CpGV-M (M), CpGV-I12 (I12), CpGV-E2 (E2), CpGV-S (S) and CpGV-I07 (I07). CpGV-M was set as the reference sequence. For CpGV-M1, only major differences to all other isolates are shown. Arrows represent the relative length, orientation and position ORFs. Color code: white = ORFs are identical in their amino acid (aa) sequence to CpGV-M, red = ORFs are unique in their aa sequence, blue = ORFs with the same aa sequence, but different to CpGV-M. Deletions and insertions are represented by orange and green triangles, respectively, with their size given below. The putative transposable elements of CpGV-I12 and CpGV-E2 are shown by purple triangles. The bottom line represents the location of 13 CpGV-dispersed repeats (black boxes), 30 repeat regions (white boxes), and the major repeat region (light grey box).

The basic characteristics of the genome sequences of different CpGV isolates are given in Table 1 and Table S1. Since CpGV-M1 was the first CpGV genotype whose genome was completely sequenced [8], the genome sequences of CpGV-M, -I12, -E2, -S, and -I07 were annotated by referring to the ORF numbers of CpGV-M1 (Figure 1). Subsequently, sequenced CpGV isolates showed a high level of co-linearity in their genomes, which was indicated by an arrangement of common ORFs in the same order, and the orientation of all five CpGV isolates (Figure 1). The maximum number of ORFs was 142 for CpGV-M and CpGV-I12 (Table 1). Each ORF was homologous for at least four out of the five isolates CpGV-M, -I12, -E2, -S and -I07 (Figure 1). Isolates with missing ORFs were CpGV-E2 (*cp26* and *cp65*), CpGV-S (*cp25* and *cp26*), and CpGV-I07 (*cp61*, *cp63*–*cp66*, *cp72*, and *cp78*) (Figure 1) which reduced the amount of detected ORFs to 140 (CpGV-E2 and CpGV-S) and 137 (CpGV-I07) (Table 1). The loss of ORF *cp25* (CpGV-S) and *cp26* (CpGV-S and -E2), both associated to a direct and major repeat region (Figure 1, Table S3), were due to a 13-bp insertion (premature stop codon) and a 38-bp deletion (loss of start codon), respectively. The missing ORFs *cp65* (CpGV-E2), *cp72*, and *cp63*–*cp66* (CpGV-I07) were due to larger deletions (>120 bp) causing premature stop codons or omitting whole ORFs (Figure 1). Only ORFs *cp61* and *cp78*, both CpGV-I12 and with a premature stop codon, were caused by a single nucleotide insertion and deletion, respectively.

However, the ORF annotation in CpGV-M, -I12, -E2, -S and -I07 faced some difficulties, since CpGV-M1 tandem ORFs *cp28* and *cp29*, *odv-e66* and *cp38*, *cp51* and *cp52*, as well as *cp129* and *cp130*, appeared to be fused, which resulted in *cp28/29*, *odv-e66*, *cp51/52*, and *cp129/130*, respectively (Figure 1, Table S1). Also, a fission of CpGV-M1 ORFs was observed in all other isolates: *cp36* split into *cp36a*/*cp36b*, and *cp52* split into *cp52a*/*cp52b* (Figure 1, Table S1). The reason for fission or fusion was single nucleotide insertions and deletions, which were only detected in CpGV-M1, and not supported by any other isolate sequence. The only exceptions were *cp50*/*cp51* and *cp120a*/*cp120b* (*dnaligase*) in CpGV-I07. Here, the reason for ORF splitting was the insertion of a single nucleotide causing a frameshift (Figure 1). With the exception of *cp120a*/*cp120b* in CpGV-I07, ORF splitting and fusion in CpGV-M1 caused ORFs that were solely unique in their amino acid sequences. The accumulation of such significant differences in only CpGV-M1, an in vivo cloned genotype of CpGV-M, is doubtful. Indeed, it could be a consequence of the in vivo cloning procedure. However, since CpGV-M1 was one of the first sequenced betabaculovirus genomes [8], reference sequences for comparison were not available for a scrutinized analysis, and it is therefore likely that minor annotation errors of the original sequencing raw data may have resulted in these inconsistencies. For that reason, for further analyses, the re-sequenced genome of CpGV-M was chosen as the reference sequence instead of CpGV-M1.

**Figure 2:**
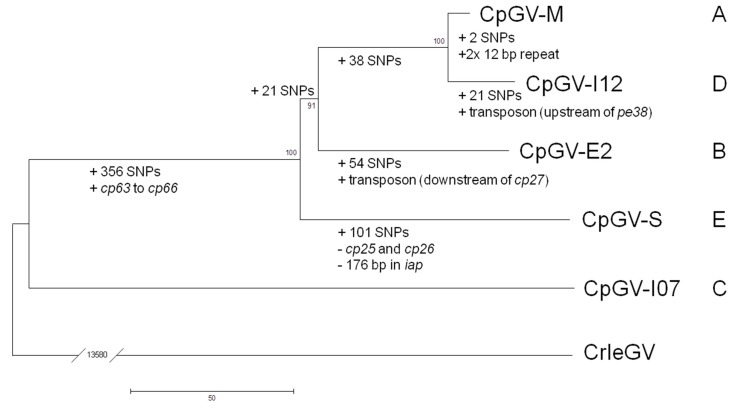
CpGV phylogeny and evolutionary trends of CpGV-M, -I12, -E2, -S and -I07. Phylogeny is based on the minimum evolution method of the alignment of whole genome nucleotide sequences with 1000 bootstrap replicates. CpGV genome groups A to E are given to the right. Cryptophlebia leucotreta granulovirus (CrleGV) was set as outgroup. Group and isolate specific single nucleotide polymorphisms (SNPs) and additional specific genomic features are given below each branch.

Another characteristic feature of CpGV genomes is the presence of 13 copies of a single inverted repeat, formerly called a dispersed repeat, for CpGV [8], which were present in all sequenced isolates (Figure 1). They might also be considered putative homologous repeat regions (*hrs*), which are a well-known feature of many baculoviruses, and are supposed to function as enhancers of gene transcription or origins of DNA replication [27,28,29]. In contrast to CpGV, where each repeat region is about 75 bp long and only contains a single unit repeat (Table S2), *hrs* usually consists of several tandem repeated sequences, and is an imperfect palindrome. The dispersed CpGV repeats share a common motif [8], and were altered only by the SNPs of some isolates (Table S2). The fully sequenced genome of CrleGV, the most closely related neighbor of CpGV [30] (Figure 2), was annotated to have three *hrs*, all in similar locations as CpGV-dispersed repeat #1 (between *cp75* and *ie-1*), #2 (downstream of *p49*) and #6 (between *sod* and *p74*) (Figure 2, Table S2), but lacked sequence homology to CpGV (data not shown).

The major repeat region of CpGV-M1 [8], spanning from *cp25* to *cp26*, was confirmed, but with variability in CpGV-M, -I12, -E2, -S and -I07. The major repeat region consists of three large repeats, which are subdivided into sections A, B, and C [8]. A significant deletion of section B and C (second large repeat) and A (third large repeat) was present in CpGV-E2 and -S. Various small deletions and insertions shortened the major repeat region by 281 bp, 233 bp, and 84 bp for CpGV-E2, -S and -I07 (Figure 1), respectively, in comparison to the total size of the 946-bp major repeat region of CpGV-M and -I12. An additional thymidine at position 21,701 (in CpGV-M) within section C (first large repeat) was only absent in CpGV-M1, which resulted in a size change for *cp26* and *cp27* (Figure 1). Besides this single nucleotide deletion in CpGV-M1, no other sequence difference in the major repeat region was observed between CpGV-M, -I12 and CpGV-M1. The presence and size of *cp25*, *cp26* and *cp27* were explained by the overall variability of the CpGV major repeat region.

A repeat region of CrleGV, called non-*hr*, was found in the same relative position as the major repeat region of CpGV, downstream of *pe38* and in the vicinity of *cp27* [30] (Figure 1).

Furthermore, another 30 repeat motifs could be found in CpGV-M, -I12, -E2, -S and -I07, which occurred successively at least twice in one of the five genomes (Figure 1, Table S3). These repeats also include the 2 × 12 bp repeat within *pe38* (*cp24*) (Table S3), which is present in CpGV-M, but not in -I12, -E2, -S and -I07. This 2 × 12 bp motif was demonstrated to be the key target of type I resistance of CM against CpGV-M [5].

Upstream of *pe38* (*cp24*), another 47-bp long repeat was found (repeat #5, Table S3) that was present once in CpGV-M, -E2 and -S, and three times in I07. In CpGV-I12, only the first 22 bp of the repeat is present, which is then followed by an insertion (Figure 1) that shows features of typical transposable elements, including inverted terminal repeats and the duplication of a putative target site [7]. Besides CpGV-I12, the presence of a putative transposable element was also shown for CpGV-I01 (GenBank accession no. EU_370251), which was slightly larger in CpGV-E2. Whereas in CpGV-I12 and -I01 the element is located upstream of *pe38*, its presence in CpGV-E2 is at a different genomic location downstream of *cp27* (Figure 1) [7]. Two putative ORFs, 108 and 129 bp in length, were found within the transposable element of CpGV-I12 and -E2, but no hits other than CpGV itself were found with BLASTn (megablast), BLASTp and PSI-BLAST (data not shown). A BLASTn search of the entire transposable elements only provided hits for CpGV itself (data not shown). The origin of the putative transposable element in CpGV-I12, -I01 and -E2 remains unclear. Stable transposon insertions from hosts such as *C. pomonella* and *Cryptophlebia leucotreta* were occasionally found in CpGV mutants after infection of larvae [31,32], and can further lead to an inversion in the CpGV genome [33]. As recently noted by ultra-deep DNA sequencing, the horizontal transfer of host transposons into baculovirus genomes seems to occur rather frequently, which supports early hypotheses that baculoviruses may play a role as vectors for horizontal transposon transfer between insects [32,34].

By phylogenetic analysis, based on concatenated partial nucleotide sequences of *polh*/*gran* (*cp1*) and *lef-8* (*cp131*), CpGV isolates were classified initially into genome groups A to D [7]. CpGV-S was assigned a further genome type, group E, which grouped it to a more basal lineage than CpGV-M and -I12, but more distal than CpGV-E2, according to a phylogenetic analysis based on the amino acid sequences of the 35 core genes of all isolates [5].

Due to the high genomic co-linearity and the void of translocations and inversions, the six nucleotide sequences of CpGV-M, -I12, -E2, -S and -I07 were aligned, and phylogenetic analysis was conducted on all of the nucleotide sequences (Figure 2). More informative positions were obtained from considering the aligned sequences of the CpGV isolates than using the amino acid sequences of highly conserved core genes alone. The result of this approach was similar to the phylogenetic tree based on core genes [5], except that CpGV-E2 and -S switched their position in the tree, which presented CpGV-E2 as the more ancestral genome group. This tree architecture is strongly supported by high bootstrap values (Figure 2).

The basic properties of the genomes varied with position in the phylogenetic tree (Figure 2). From the most basal (CpGV-I07) to the most distal lineages (CpGV-M/-I12), genomes increased in size (CpGV-I07: 120,816 bp to CpGV-M/-I12: 123,500 bp; the sizes of transposable elements, 715 bp and 684 bp for CpGV-E2 and CpGV-I12, respectively, were not considered), contained a higher number of ORFs (CpGV-I07: 137 ORFs to CpGV-M/-I12: 142 ORFs), and exhibited a reduced GC content (CpGV-I07: 45.39% to CpGV-I12: 45.21%) (Table 1). For the latter, CpGV-M appeared to be the only exception, since its GC content (45.27%) was found to be almost similar to CpGV-S (45.28%) (Table 1). Possible correlations between a genome size and the GC content of CpGV-M are unknown and remain speculative.

**Figure 3:**
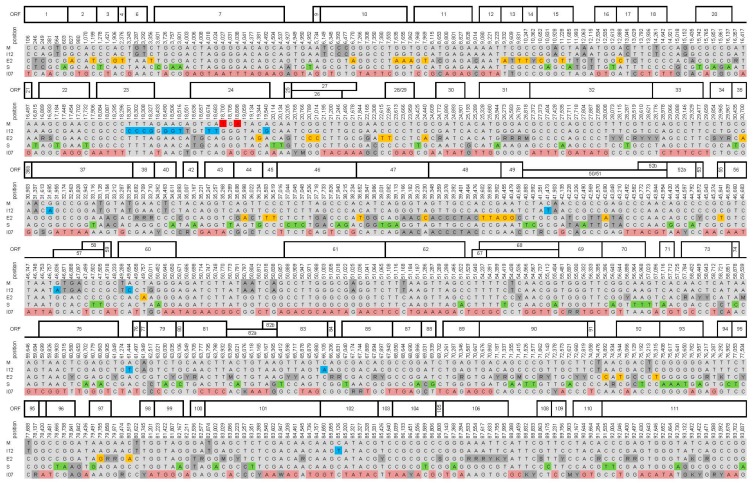

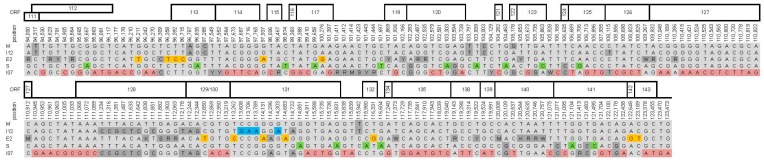
Alignment of all 788 SNP positions covered by all five CpGV isolates. Positions refer to the reference sequence CpGV-M, as well as the ORF numbers on top of the alignment. Red: SNPs specific for CpGV-M (=2), blue: SNPs specific for CpGV-I12 (=21), orange: SNPs specific for CpGV-E2 (=54), green: SNPs specific for CpGV-S (=101), light red: SNPs specific for CpGV-I07 (=356), dark grey: unspecific (=116) and ambiguous (=137) SNP positions, light grey = SNP shared by three or four isolates. Position 20,144 was specific for CpGV-S and CpGV-I07. Ambiguities: R (A or G), Y (C or T), S (G or C), W (A or T), M (A or C) and K (G or T).

Considering sequence ambiguities, it was found that the isolates CpGV-M, -I12 and -S were highly homogenous, whereas “clone” CpGV-E2 and isolate CpGV-I07 showed some ambiguities in their consensus sequence, which hinted at some heterogeneity in these specimens (Figure 3). These ambiguous nucleotide positions were not included in the following SNP analysis. With 356 specific SNPs, the isolate CpGV-I07 possessed the highest number of unique SNP positions, which discriminated it from CpGV-M, -I12, -E2 and -S. The number of unique SNP positions of CpGV-S (101 SNPs), -E2 (54 SNPs), -I12 (21 SNPs), and -M (only two unique SNPs) correlated with the branch lengths of the whole genome sequence tree (Figure 2). This correlation supports our conclusion of a slow evolution of CpGV-M compared with CpGV-I12 and other isolates (Table 1, Figure 3). Besides the two and 21 unique SNP positions of CpGV-M and CpGV-I12, respectively, both isolates shared a set of 38 additional positions, which underlined the close relatedness of both isolates (Figure 2). Further sets of branch-specific SNP positions were found for CpGV-M/I12 (38 SNPs), CpGV-M/I12/E2 (21 SNPs), CpGV-M/I07 (1 SNP), CpGV-I12/I07 (13 SNPs), CpGV-E2/S (20 SNPs), and CpGV-E2/I07 (22 SNPs) (Figure 2 and Figure 3). Only one SNP position (CpGV-M: 20,114) was detected to encode for three different nucleotides (Figure 3). In general, SNP positions were located all over the genomes, but were twice as likely in non-coding and repeat regions (0.012 SNPs/bp) than in coding sequences (0.006 SNPs/bp) (Figure 3). In repeat regions, SNP identification could be ambiguous depending on the applied alignment parameters, which have an effect on how repeats in combination with incomplete repeats are aligned. Therefore, an isolate-identifying method such as qPCR-based SNP detection and sequencing, should consider unambiguous regions.

In conclusion, the genome sequences of the isolates CpGV-M, -I12, -E2, -S and -I07, with different geographic origins and belonging to different genome groups (A to E,) comprise the current known genetic diversity of CpGV. Hence, the isolate group-specific SNPs may be useful for the identification of present genome groups in newly discovered isolates.

## Figures and Tables

**Table 1:** General Cydia pomonella granulovirus (CpGV) genome features.

Genome Feature	CpGV Isolate				
	M	I12	E2	S	I07
sequencing method	454	Sanger	454	454	454
average coverage	17.23	3.9	243	22.21	8.05
genome length (bp)	123,529	124,269	123,858	123,193	120,816
coding (bp)	113,121	113,145	111,822	111,372	109,635
(% of total size)	(91.57)	(91.05)	(90.28)	(90.40)	(90.75)
non-coding (bp)	10,408	11,124	12,036	11,821	11,181
(% of total size)	(8.43)	(8.95)	(9.72)	(9.60)	(9.25)
GC%	45.27	45.21	45.21	45.28	45.39
number of open reading frames (ORF)	142	142	140	140	137
ORFs with identical amino acid (aa) compared to CpGV-M		137	77	76	48

## Supplementary Materials

The following are available online at www.mdpi.com/1999-4915/9/8/227/s1, Table S1: Open reading frames (ORFs) and their positions within isolates of the Cydia pomonella granulovirus, Table S2: CpGV dispersed repeats (DR) of CpGV-M as found in CpGV-M1, -I12, -E2, -S and -I07, Table S3: Repeats of the CpGV genome.
