# Supplementary material for: Deciphering Single Nucleotide Polymorphisms and Evolutionary Trends in Isolates of the Cydia pomonella granulovirus
Source: Viruses. 2017 Aug 18;9(8):227. doi: 10.3390/v9080227 (PMC5580484; doi:10.3390/v9080227)
Supplement: Supplementary file 1 [file viruses-09-00227-s001.pdf]

Supplementary material

Table S1. Open reading frames (ORFs) and their positions within isolates of the Cydia pomonella granulovirus (CpGV). Each row represents one homologue ORF with its name and description to the left. Arrows (</>) represent ORF orientation. Promoter motifs were searched within 120 nt upstream of ORFs. Early gene (e) promoters were identified by TATA box and CAGT motif, at which transcription s initiated (van Oers & Vlak, 2007). Late and very late (l) promoter motifs were characterized by a (A/T/G)TAAG motif (Xu et al., 1995).

ORF	description	CpGV-M				CpGV-I12				CpGV-E2				CpGV-S				CpGV-I07			
			Min		Max		Min		Max		Min		Max		Min		Max		Min		Max
cp1	granulin	l	1	>	747	l	1	>	747	l	1	>	747	l	1	>	747	l	1	>	747
cp2		e, l	749	<	1,273	e, l	749	<	1,273	e, l	749	<	1,273	e, l	749	<	1,276	e, l	749	<	1,279
cp3	pk-1	e	1,254	>	2,093	e	1,254	>	2,093	e	1,254	>	2,093	e	1,257	>	2,096	e	1,260	>	2,021
cp4		e, l	2,173	<	2,739	e, l	2,173	<	2,739	e, l	2,173	<	2,739	e, l	2,175	<	2,741	e, l	2,180	<	2,746
cp5			2,729	>	2,971		2,729	>	2,971		2,729	>	2,971		2,731	>	2,973		2,736	>	2,978
cp6		l	3,122	>	3,298	l	3,122	>	3,298	l	3,122	>	3,298	l	3,124	>	3,327	l	3,129	>	3,278
cp7	ie-1	e	3,391	<	4,857	e	3,391	<	4,857	e	3,431	<	4,897	e	3,420	<	4,886	e	3,340	<	4,833
cp8		e	4,963	>	5,541	e	4,963	>	5,541	e	5,002	>	5,580	e	4,992	>	5,570	e	4,939	>	5,517
cp9		e, l	5,581	<	5,886	e, l	5,581	<	5,886	e, l	5,620	<	5,925	e, l	5,610	<	5,915	e, l	5,558	<	5,863
cp10	chitinase	e	6,027	<	7,811	e	6,027	<	7,811	e	6,066	<	7,850	e	6,055	<	7,839	e	6,004	<	7,788
cp11	cathepsin	l	7,934	>	8,935	l	7,934	>	8,935	l	7,955	>	8,956	l	7,962	>	8,963	l	7,904	>	8,908
cp12		l	9,015	>	9,248	l	9,015	>	9,248	l	9,036	>	9,269	l	9,043	>	9,276	l	8,988	>	9,227
cp13	gp37	l	9,318	<	10,073	l	9,318	<	10,073	l	9,352	<	10,095	l	9,348	<	10,103	l	9,294	<	10,049
cp14	odv-e18	l	10,145	<	10,399	l	10,146	<	10,400	l	10,181	<	10,435	l	10,232	<	10,486	l	10,107	<	10,361
cp15	p49	e, l	10,400	<	11,773	e, l	10,401	<	11,774	e, l	10,436	<	11,809	e, l	10,487	<	11,860	e, l	10,362	<	11,735
cp16		e	12,145	<	12,735	e	12,146	<	12,736	e	12,178	<	12,768	e	12,248	<	12,877	e	12,124	<	12,717
cp17	iap-3	e	12,865	>	13,692	e	12,866	>	13,693	e	12,898	>	13,725	e	12,969	>	13,796	e	12,848	>	13,675
cp18	odv-e56	e, l	13,730	<	14,797	e, l	13,731	<	14,798	e, l	13,763	<	14,830	e, l	13,834	<	14,901	e, l	13,713	<	14,780
cp19	orf15R	e	15,171	>	15,398	e	15,172	>	15,399	e	15,204	>	15,431	e	15,275	>	15,502	e	15,156	>	15,383
cp20	orf16L	l	15,458	<	16,165	l	15,459	<	16,166	l	15,491	<	16,198	l	15,562	<	16,260	l	15,443	<	16,150
cp21	orf17L		16,434	<	16,613		16,435	<	16,614		16,491	<	16,670		16,529	<	16,708		16,419	<	16,598
cp22	orf17R	l	16,836	>	17,879	l	16,837	>	17,880	l	16,893	>	17,936	l	16,925	>	17,968	l	16,820	>	17,863
cp23		e, l	17,970	>	18,428	e, l	17,971	>	18,429	e, l	18,027	>	18,485	e, l	18,059	>	18,517	e, l	17,954	>	18,412
cp24	pe-38	e	18,571	<	19,719	e	19,314	<	20,438	e	18,624	<	19,748	e	18,661	<	19,785	e	18,651	<	19,775
cp25		e	20,165	<	20,329	e	20,884	<	21,048	e	20,132	<	20,266					e	20,131	<	20,307
cp26		l	20,328	>	21,314	l	21,047	>	22,033									l	20,306	>	21,208
cp27		e	20,358	<	21,827	e	21,077	<	22,546		20,320	<	21,546	e	20,370	<	21,644	e	20,336	<	21,721
cp28/29		e	22,688	>	24,058	e	23,407	>	24,777	e	23,116	>	24,486	e	22,505	>	23,875	e	22,582	>	23,952
cp30		e	24,629	>	25,174	e	25,348	>	25,893	e	24,995	>	25,540	e	24,399	>	24,944	e	24,443	>	24,706
cp31	F-protein	e	25,306	>	27,099	e	26,025	>	27,818	e	25,673	>	27,466	e	25,088	>	26,881	e	25,086	>	26,915
cp32		l	27,325	>	28,665	l	28,044	>	29,384	l	27,692	>	29,107	l	27,107	>	28,474	l	27,148	>	28,491
cp33		l	28,737	<	29,621	l	29,456	<	30,340	l	29,179	<	30,063	l	28,546	<	29,430	l	28,563	<	29,447
cp34		e	29,664	<	30,353	e	30,383	<	31,072	e	30,106	<	30,795	e	29,567	<	30,181	e	29,490	<	30,179
cp35	pif-3	e, l	30,247	>	30,822	e, l	30,966	>	31,541	e, l	30,689	>	31,264	e, l	30,075	>	30,650	e, l	30,073	>	30,648
cp36b	orf36b	e	30,901	<	31,083	e	31,620	<	31,802	e	31,344	<	31,526	e	30,730	<	30,912	e	30,765	<	30,908
cp36a	orf36a	e	30,998	<	31,162	e	31,717	<	31,881	e	31,441	<	31,605	e	30,827	<	30,991	e	30,823	<	30,987
cp37	odv-e66	l	31,205	<	33,439	l	31,924	<	34,158	l	31,647	<	33,914	l	31,033	<	33,267	l	31,029	<	33,287
cp39		l	33,481	>	33,798	l	34,200	>	34,517	l	33,958	>	34,275	l	33,309	>	33,626	l	33,329	>	33,646
cp40		e	33,851	<	34,180	e	34,570	<	34,899	e	34,336	<	34,665	e	33,687	<	34,016	e	33,699	<	34,028
cp41	lef-2	e	34,321	>	34,836	e	35,040	>	35,555	e	34,806	>	35,321	e	34,157	>	34,672	e	34,169	>	34,684

cp42	orf35Ra	e	34,928	>	35,176	e	35,647	>	35,895	e	35,413	>	35,661	e	34,764	>	35,012	e	34,776	>	35,024
cp43		e, l	35,223	<	35,567	e, l	35,942	<	36,286	e, l	35,708	<	36,052	e, l	35,059	<	35,403	e, l	35,075	<	35,422
cp44	orf36L	e, l	35,632	<	36,255	e, l	36,351	<	36,974	e, l	36,117	<	36,740	e, l	35,468	<	36,091	e, l	35,487	<	36,110
cp45		e	36,314	<	36,778	e	37,033	<	37,497	e	36,799	<	37,263	e	36,150	<	36,614	e	36,169	<	36,633
cp46	mp-nase		36,836	<	38,473		37,555	<	39,192		37,321	<	38,958		36,672	<	38,312		36,691	<	38,328
cp47	p13	l	38,480	>	39,289	l	39,199	>	40,008	l	38,965	>	39,777	l	38,319	>	39,128	l	38,335	>	39,147
cp48	pif-1		39,333	>	40,451		40,052	>	41,170		39,797	>	40,915		39,172	>	40,290		39,167	>	40,285
cp49		l	40,448	<	40,837	l	41,167	<	41,556	l	40,912	<	41,319	l	40,287	<	40,676	l	40,282	<	40,671
cp50																		l	40,607	>	42,469
cp51																			42,471	>	43,619
cp50/51		l	40,773	>	44,090	l	41,492	>	44,794	l	41,255	>	44,608	l	40,612	>	43,929				
cp52b	orf52b	e	43,709	<	44,071	e	44,413	<	44,775	e	44,224	<	44,589	e	43,545	<	43,910	e	43,491	<	43,877
cp52a	orf52a	l	44,099	<	44,839	l	44,803	<	45,543	l	44,617	<	45,357	l	43,938	<	44,678	l	43,905	<	44,645
cp53		e	44,849	>	44,995	e	45,553	>	45,699	e	45,367	>	45,513	e	44,688	>	44,834	e	44,655	>	44,801
cp54	ubiquitin		45,071	<	45,355		45,775	<	46,059		45,589	<	45,873		44,910	<	45,194		44,877	<	45,179
cp55	odv-ec43	e, l	45,434	>	46,498	e, l	46,138	>	47,202	e, l	45,952	>	47,016	e, l	45,273	>	46,337	e, l	45,258	>	46,322
cp56		l	46,505	>	46,714	l	47,209	>	47,418	l	47,023	>	47,232	l	46,344	>	46,553	l	46,329	>	46,538
cp57	pp31/39K	e	46,791	<	47,516	e	47,495	<	48,220	e	47,309	<	48,034	e	46,630	<	47,373	e	46,617	<	47,339
cp58	lef-11		47,467	<	47,871		48,171	<	48,575		47,985	<	48,389		47,306	<	47,728		47,293	<	47,694
cp59	sod	l	47,826	<	48,224	l	48,530	<	48,928	l	48,344	<	48,817	l	47,683	<	48,156	l	47,649	<	48,122
cp60	p74	l	48,598	<	50,664	l	49,302	<	51,368	l	49,120	<	51,186	l	48,458	<	50,524	l	48,425	<	50,491
cp61		l	50,883	<	51,095	l	51,587	<	51,799	l	51,404	<	51,616	l	50,742	<	50,954	l			
cp62		e, l	51,067	<	51,636	e, l	51,771	<	52,376	e, l	51,588	<	52,097	e, l	50,926	<	51,435	e, l	50,916	<	51,356
cp63	bro	e	51,774	>	51,941	e	52,514	>	52,681	e	52,213	>	52,380	e	51,573	>	51,740				
cp64		e, l	52,654	>	53,346	e, l	53,394	>	54,086	e, l	53,095	>	53,808	e, l	52,675	>	53,367				
cp65		l	53,444	<	53,683	l	54,184	<	54,423					l	53,465	<	53,704				
cp66			53,838	>	54,059		54,578	>	54,799		54,171	>	54,392		53,859	>	54,098				
cp67		l	54,134	<	54,397	l	54,874	<	55,137	l	54,467	<	54,730	l	54,150	<	54,413	l	51,402	<	51,665
cp68	p47	e	54,369	>	55,751	e	55,109	>	56,491	e	54,702	>	56,084	e	54,385	>	55,767	e	51,493	>	52,689
cp69		l	55,790	>	56,452	l	56,530	>	57,192	l	56,123	>	56,785	l	55,806	>	56,468	l	53,063	>	53,614
cp70		l	56,526	<	57,080	l	57,266	<	57,820	l	56,859	<	57,413	l	56,541	<	57,116	l	53,801	<	54,379
cp71	p24	l	57,150	>	57,761	l	57,890	>	58,501	l	57,483	>	58,094	l	57,178	>	57,789	l	54,447	>	55,058
cp72		e	57,800	<	58,144	e	58,540	<	58,884	e	58,133	<	58,477	e	57,828	<	58,103	e	55,513	<	56,115
cp73	38.7kd	e	58,338	<	58,934	e	59,078	<	59,674	e	58,671	<	59,267	e	58,218	<	58,814	e			
cp74	lef-1	e	58,915	<	59,622	e	59,655	<	60,362	e	59,248	<	59,955	e	58,795	<	59,502	e	56,096	<	56,803
cp75	pif-2	e, l	59,748	>	61,364	e, l	60,488	>	62,104	e, l	60,081	>	61,697	e, l	59,628	>	61,244	e, l	56,929	>	58,545
cp76	fgf	e	61,523	<	62,203	e	62,265	<	62,945	e	61,856	<	62,536	e	61,403	<	62,083	e	58,711	<	59,391
cp77		e	62,271	<	62,582	e	63,013	<	63,324	e	62,604	<	62,915	e	62,151	<	62,456	e	59,459	<	59,764
cp78		e	62,591	<	62,818	e	63,333	<	63,560	e	62,924	<	63,151	e	62,465	<	62,692	e			
cp79		l	62,842	>	63,312	l	63,584	>	64,054	l	63,175	>	63,645	l	62,716	>	63,186	l	60,023	>	60,493
cp80	lef-6	e	63,309	<	63,614	e	64,051	<	64,356	e	63,642	<	63,947	e	63,183	<	63,488	e	60,490	<	60,795
cp81	dbp	e	63,693	<	64,565	e	64,435	<	65,307	e	64,026	<	64,898	e	63,567	<	64,439	e	60,874	<	61,746
cp82b	orf82b	e	64,592	<	64,849	e	65,334	<	65,591	e	64,925	<	65,182	e	64,647	<	65,339	e	61,954	<	62,646
cp82a	orf82a	l	64,773	<	65,465	l	65,515	<	66,207	l	65,106	<	65,798	l	64,466	<	64,723	l	61,773	<	62,030
cp83	p45	e	65,355	>	66,674	e	66,097	>	67,416	e	65,688	>	67,007	e	65,229	>	66,548	e	62,536	>	63,855
cp84	p12	l	66,706	>	67,035	l	67,448	>	67,777	l	67,039	>	67,368	l	66,580	>	66,909	l	63,887	>	64,216
cp85	odv-c42	e, l	67,094	>	68,236	e, l	67,836	>	68,978	e, l	67,427	>	68,569	e, l	66,968	>	68,110	e, l	64,275	>	65,417
cp86	p6.9	l	68,268	>	68,417	l	69,010	>	69,159	l	68,601	>	68,750	l	68,142	>	68,291	l	65,449	>	65,598
cp87	lef-5		68,518	<	69,246		69,260	<	69,988		68,851	<	69,579		68,392	<	69,120		65,699	<	66,427
cp88	38K		69,070	>	70,101		69,812	>	70,843		69,403	>	70,434		68,944	>	69,975		66,251	>	67,282
cp89		l	70,269	<	70,754	l	71,011	<	71,496	l	70,607	<	71,092	l	70,143	<	70,628	l	67,455	<	67,940
cp90	helicase	l	70,738	>	74,133	l	71,480	>	74,875	l	71,076	>	74,471	l	70,612	>	74,007	l	67,924	>	71,313
cp91	odv-e25	e, l	74,245	<	74,886	e, l	74,987	<	75,628	e, l	74,585	<	75,226	e, l	74,119	<	74,760	e, l	71,429	<	72,070

cp92		l	74,976	<	75,461	l	75,719	<	76,204	l	75,316	<	75,801	l	74,850	<	75,335	l	72,160	<	72,645
cp93	p33	e, l	75,525	>	76,280	e, l	76,268	>	77,023	e, l	75,865	>	76,620	e, l	75,399	>	76,154	e, l	72,709	>	73,464
cp94	iap	l	76,324	<	77,055	l	77,067	<	77,798	l	76,664	<	77,395	l	76,424	<	76,705	l	73,508	<	74,239
cp95	lef-4	l	77,060	<	78,502	l	77,803	<	79,245	l	77,400	<	78,842	l	76,758	<	78,200	l	74,244	<	75,686
cp96	vp39	l	78,574	>	79,431	l	79,317	>	80,174	l	78,914	>	79,771	l	78,272	>	79,129	l	75,758	>	76,615
cp97	odv-ec27	e, l	79,573	>	80,439	e, l	80,316	>	81,182	e, l	79,913	>	80,779	e, l	79,271	>	80,137	e, l	76,757	>	77,623
cp98	ptp-2	e	80,753	>	81,238	e	81,496	>	81,981	e	81,093	>	81,578	e	80,451	>	80,936	e	77,937	>	78,422
cp99		e	81,329	<	82,513	e	82,072	<	83,256	e	81,669	<	82,853	e	81,027	<	82,211	e	78,490	<	79,674
cp100		l	82,545	>	82,871	l	83,288	>	83,614	l	82,885	>	83,211	l	82,243	>	82,569	l	79,706	>	80,032
cp101	vp91	e, l	82,906	<	84,903	e, l	83,649	<	85,646	e, l	83,246	<	85,243	e, l	82,604	<	84,601	e, l	80,122	<	82,119
cp102	tlp20	l	84,884	>	85,534	l	85,627	>	86,277	l	85,224	>	85,874	l	84,582	>	85,232	l	82,100	>	82,750
cp103		l	85,509	>	86,084	l	86,252	>	86,827	l	85,849	>	86,424	l	85,207	>	85,782	l	82,725	>	83,300
cp104	gp41	l	86,110	>	86,979	l	86,853	>	87,722	l	86,450	>	87,319	l	85,808	>	86,677	l	83,326	>	84,195
cp105		l	87,059	>	87,319	l	87,802	>	88,062	l	87,399	>	87,659	l	86,757	>	87,017	l	84,287	>	84,547
cp106	vlf-1		87,276	>	88,412		88,019	>	89,155		87,616	>	88,752		86,974	>	88,110		84,504	>	85,640
cp107		e, l	88,508	>	88,762	e, l	89,251	>	89,505	e, l	88,848	>	89,102	e, l	88,205	>	88,459	e, l	85,735	>	85,989
cp108		l	88,874	>	89,320	l	89,617	>	90,063	l	89,214	>	89,660	l	88,571	>	89,017	l	86,101	>	86,547
cp109			89,415	<	89,990		90,158	<	90,733		89,755	<	90,330		89,112	<	89,699		86,661	<	87,236
cp110		e	90,352	<	90,729	e	91,064	<	91,471	e	90,647	<	91,054	e	90,016	<	90,423	e	87,575	<	87,961
cp111	dnapol		90,849	<	94,004		91,591	<	94,746		91,174	<	94,329		90,543	<	93,698		88,081	<	91,236
cp112	desmoplakin	e	93,946	>	96,102	e	94,688	>	96,844	e	94,271	>	96,430	e	93,640	>	95,793	e	91,178	>	92,908
cp113	lef-3	e	96,313	<	97,374	e	97,055	<	98,116	e	96,642	<	97,703	e	96,004	<	97,065	e	93,538	<	94,599
cp114		e	97,343	>	97,723	e	98,085	>	98,465	e	97,672	>	98,052	e	97,034	>	97,414	e	94,568	>	94,948
cp115		e	97,845	>	98,351	e	98,587	>	99,093	e	98,174	>	98,680	e	97,555	>	98,061	e	95,072	>	95,578
cp116	iap-5	e	98,533	>	99,360	e	99,275	>	100,102	e	98,862	>	99,689	e	98,243	>	99,070	e	95,760	>	96,587
cp117	lef-9	l	99,335	>	100,834	l	100,077	>	101,576	l	99,664	>	101,163	l	99,045	>	100,544	l	96,562	>	98,061
cp118	fp25K	l	100,870	>	101,355	l	101,612	>	102,097	l	101,199	>	101,684	l	100,580	>	101,065	l	98,097	>	98,582
cp119		e	101,468	>	101,956	e	102,210	>	102,698	e	101,797	>	102,285	e	101,178	>	101,666	e	98,695	>	99,183
cp120	dnaligase		102,007	<	103,719		102,749	<	104,461		102,336	<	104,048		101,698	<	103,410				
cp120a																			99,772	<	100,947
cp120b																			99,234	<	99,821
cp121			104,019	>	104,234		104,761	>	104,976		104,348	>	104,563		103,710	>	103,925		101,247	>	101,462
cp122		e	104,328	>	104,528	e	105,070	>	105,270	e	104,657	>	104,857	e	104,019	>	104,219	e	101,556	>	101,753
cp123	fgf		104,618	<	105,820		105,360	<	106,562		104,947	<	106,149		104,309	<	105,511		101,842	<	103,044
cp124		e	105,973	>	106,272	e	106,716	>	107,015	e	106,303	>	106,602	e	105,664	>	105,963	e	103,197	>	103,496
cp125	alk-exo	e	106,435	>	107,631	e	107,178	>	108,374	e	106,765	>	107,961	e	106,126	>	107,322	e	103,660	>	104,856
cp126	helicase-2	e	107,555	>	108,928	e	108,298	>	109,671	e	107,885	>	109,258	e	107,246	>	108,619	e	104,780	>	106,153
cp127	rr1	e	109,017	<	110,891	e	109,760	<	111,634	e	109,347	<	111,221	e	108,708	<	110,582	e	106,242	<	108,185
cp128	rr2a		111,019	>	112,107		111,762	>	112,850		111,349	>	112,437		110,710	>	111,798		108,275	>	109,363
cp129/130		e	112,140	<	113,120	e	112,883	<	113,860	e	112,470	<	113,450	e	111,831	<	112,811	e	109,396	<	110,385
cp131	lef-8	e	113,198	<	115,819	e	113,938	<	116,559	e	113,528	<	116,149	e	112,889	<	115,510	e	110,466	<	113,090
cp132			115,907	>	116,302		116,647	>	117,042		116,236	>	116,631		115,597	>	115,992		113,177	>	113,575
cp133		e	116,364	<	116,552	e	117,104	<	117,292	e	116,693	<	116,881	e	116,054	<	116,242	e	113,637	<	113,825
cp134		l	116,542	>	116,943	l	117,282	>	117,683	l	116,871	>	117,272	l	116,232	>	116,633	l	113,815	>	114,216
cp135		l	117,012	<	118,142	l	117,752	<	118,882	l	117,341	<	118,471	l	116,702	<	117,832	l	114,285	<	115,427
cp136		l	118,148	<	118,369	l	118,888	<	119,109	l	118,477	<	118,698	l	117,838	<	118,059	l	115,433	<	115,654
cp137	lef-10	e, l	118,320	>	118,589	e, l	119,060	>	119,329	e, l	118,649	>	118,918	e, l	118,010	>	118,279	e, l	115,605	>	115,874
cp138	vp1054	e	118,447	>	119,445	e	119,187	>	120,185	e	118,776	>	119,774	e	118,137	>	119,135	e	115,732	>	116,730
cp139		e	119,335	<	119,655	e	120,075	<	120,395	e	119,664	<	119,984	e	119,025	<	119,345	e	116,620	<	116,940
cp140	fgf-3	e	119,756	>	120,799	e	120,496	>	121,539	e	120,085	>	121,128	e	119,446	>	120,489	e	117,041	>	118,084
cp141	egt	e	120,882	<	122,336	e	121,622	<	123,076	e	121,211	<	122,665	e	120,546	<	122,000	e	118,167	<	119,603
cp142		e	122,354	>	122,557	e	123,094	>	123,297	e	122,683	>	122,886	e	122,018	>	122,221	e	119,640	>	119,843
cp143	me53	e	122,530	>	123,441	e	123,270	>	124,181	e	122,859	>	123,770	e	122,194	>	123,105	e	119,816	>	120,736

Table S2. CpGV dispersed repeats (DR) of CpGV-M as found in CpGV-M1, -I12, -E2, -S and -I07. Start and end nucleotides refer to the genomic position of CpGV-M. SNP positions and indels are written below the DR sequence of CpGV-M.

Repeat No.	Isolate	Start (CpGV-M)	Sequence / Differences to CpGV-M	End (CpGV-M)	Size (bp)
1	all	3,042	ACGAGTCTGAGTTAATTTGGGCAATTTGAGAAAAATTTAAAAATTTACTTTTTCTCCCAATTAACTCGGACTCAT	3,116	75
2	M/I12	3,260	ACGAGTCCGACTTT-ATGGGCCAGAATCGGAAATTTTTAAATTTTTTACTTTTTCTCTCAATAAAGTCAGACTCGT	3,334	75
	M1		T G T		
	E2/S/I07		A A		
3	M/M1/I12/E2/S	11,903	ACGAGTCTGGCTTTATTCAAGAAATTTTAGCGAAAAACTTTTTTTCG-CTAAAATCTCGGCGCAAAGCCAGATTCGT	11,978	76
	I07		T		
4	M/I12/I07	12,068	ACGAGGCCGACTTTATTAGCTGATTTAGGAAAAATTTTAAAATTTTTAAATTTTGCCGAATAAACTCAGCCTCGT	12,142	75
	M1		-		
	E/S		T		
5	M/M1/I12/E2/S	28,918	ACGAGTCCGACTTCATCATTTGGAGATTCTGAAAATTTAAAATTTTTCTCTTTTTCTCGGATTAACTCGGACTCGT	28,993	76
	I07		C		
6	all	48,500	ACGAGTCTGGTTTTGTGCCGAGATTCTGGCGAGAAAAAGTTTTTCGCTTAAATTTTTTGAATAAAGCCAGACTCGT	48,575	76
7	M/M1/I12/E2/S	50,891	ACGAGTCTGCTTTTTTTCCAGAAATTTTAGCTAAAAACTTTTTCTCGGCAAAATCTTGGCGCAAAACCAGACTCGT	50,996	76
	I07		- A C		
8	M/M1/E2/S/I07	61,411	ACGAGTCCGAGTTTATTGGGAGAAAAAGTAAAAATTTT-AAATTTTTCTAAAAATCTCCGGATAAAGTCGGACTCGT	61,486	76
	I12		T		
9	all	77,581	ACGAGTCCGAGTTCAATCGGCAAAATCTAAAAATTTTAAAATTTTTACTTTTTCTCCCAGTTAACTCGGACTCGT	77,655	75
10	M/M1/I12/E2/S	83,891	ACGAGTCCGACTTTAGTCGGCAAAATTTTAGAAAAACTTTTTTCGTCGGAGATTCGGCGAATAAAGTCGGACTCGT	83,966	76
	I07		W		
11	M/M1/I12/E2/S	96,223	ACGAGTCCGAGTTAATTTAGCAAAAAACTAAAAATTTTAAAATTTTT--CTAAAATTTCATAATTAACTCAGACTCGT	96,298	76
	I07		A ACT		
12	M/M1/I12	103,750	ACGAGTCCGAGTTTATTATGAGATTTTAGAAAAATTTTAAATTTTTCTCTTTTCTCGTTATTAACTCGGACTCGT	103,824	75
	E2/S/I07		C		
13	all	114,594	ACGAGTCCGACTTTATTCGGAGATTTTTTAGAAAAATTTAAAATTTTTACTTTTTCACCCGTTAACTCGGACTCGT	114,669	76

Table S3. Repeats of the CpGV genome. Motifs listed are repeated successively at least twice in one of the five genomes of CpGV-M, -I12, -E2, -S, -I07. Given positions correspond to CpGV-M.

position	location	length (bp)	motif	Note
3,188...	*cp6*	27	AAATTTTACACAAGTTTAATGTACTAC	
3,322...		40	AAGTCAGACTCGTCCTAAATTTGTGAATTCTATACGAATT	overlapping with dispersed repeat 2
10,076…		14	ACACAAATTAAACA	
16,272...		24	ACATACAAAAATATCATGTTACAT	
18,428...		47	GGAAAGTAAAATATATGTATGATTTTTTTATTATTCCGGCTAGTTTA	I12 with 733 bp insertion within incomplete repeat; repeat in S with missing central TTTTTT
18,745...	*pe38*	12	TGTGTCATCCAC	key role in CM resistance against M
19,072...	*pe38*	21	CTGCTTCTCCTGCTCGAACTG	
19,830...		15	ACTGATTATGATAAA	
20,145...	*cp25*	19	ATTATAATACATTTTTATA	
20,365...	*cp2/cp27*	27	TCATCTTCATCATCAATAATGTCATCA	
22,177...		14	ACGCCCGGTCACGT	
23,465...	*cp28/29*	21	CGCCGATCTACGTCATCATTA	
24,307...		18	TTTAATTGTAAGACTTAA	
24,501...		17	AACATTTTCTACGTAAT	
27,364...	*cp32*	24	TTGGAGGCGCTGAATGCGGAGGGT	
29,772...	*cp34*	18	GCTTCGTATTAGCACTTC	
33,098...	*odv-e66*	12	GGTGGTGGAGTA	ACN CCN CCN CCN motif resulting in TPPP amino acid repeat
43,496...	*cp50/51*	15	ACCACCAGTTACTCA	
45,044...		14	TATTCTACACAATT	
51,137...	*cp62*	15	TTGTGTGGTTCATCC	
51,184...	*cp62*	18	ATCAACTCTGGGTTC	
52,662...	*cp64*	15	AGAAAGTTTGAATCC	not present in I07 (deleted region); *cp64*
56,870...	*cp70*	18	CATCAGAGTATTCGGATA	
56,940...	*cp70*	18	TTCATCCTCTGATTGGTA	
82,887...	*vp9*	55	AGAGTATGTTTTGTAACTATTAAACAGGAACAGTGAACAAACACGAGTAATCTCC	overlapping with *vp91* (*cp101*)
89,383...		15	AATAACATGGTGTTT	
89,968...	*cp109*	12	GGATTTTTCACA	with one ambiguity allowed motif is repeated more often all over the genome
97,802...		19	GGAGTTGGTTTTGTCCATC	
110,962		15	T(T/C)CGGTCAGATAA(A)CA	repeated imperfectly in I07 only as inserted duplicate
112,284...	*cp129/130*	15	TTTTATACTGCTACT	
